# Anti-metastatic effect of metformin via repression of interleukin 6-induced epithelial–mesenchymal transition in human colon cancer cells

**DOI:** 10.1371/journal.pone.0205449

**Published:** 2018-10-11

**Authors:** Sanghee Kang, Bo Ram Kim, Myoung-Hee Kang, Dae-Young Kim, Dae-Hee Lee, Sang Cheul Oh, Byung Wook Min, Jun Won Um

**Affiliations:** 1 Department of Surgery, Korea University Guro Hospital, Korea University College of Medicine, Seoul, Republic of Korea; 2 Department of Oncology, Korea University Guro Hospital, Seoul, Republic of Korea; 3 ASAN Medical Center, Department of Convergence Medicine, University of Ulsan College of Medicine, Seoul, Republic of Korea; 4 Graduate School of Medicine, Korea University College of Medicine, Seoul, Republic of Korea; 5 Department of Surgery, Korea University Ansan Hospital, Korea University College of Medicine, Gyeonggi-do, Republic of Korea; University of Navarra, SPAIN

## Abstract

Metformin, a first-line drug used to treat type 2 diabetes, has also been shown to have anticancer effects against a variety of malignancies, including colorectal cancer. Although inhibition of the mTOR pathway is known to be the most important mechanism for the antitumor effects of metformin, other mechanisms remain unclear. The purpose of this study was to identify the antitumor mechanism of metformin in colorectal cancer using high-throughput data, and then test the mechanism experimentally. We identified the gene signature of metformin-treated colon cancer cells. This signature was processed for prediction using colon adenocarcinoma patient data from the Cancer Genome Atlas to classify the patients showing a gene expression pattern similar to that in metformin-treated cells. This patient group showed better overall and disease-free survival. Furthermore, pathway analysis revealed that the metformin-predicted group was characterized by decreased interleukin (IL)-6 pathway signaling, epithelial–mesenchymal transition, and colon cancer metastatic signaling. We induced epithelial–mesenchymal transition in colon cancer cell lines via IL-6 treatment, which increased cell motility and promoted invasion. However, these effects were blocked by metformin. These findings suggest that blockade of IL-6-induced epithelial–mesenchymal transition is an antitumor mechanism of metformin.

## Introduction

Colorectal cancer has one of the highest mortality rates of all cancers worldwide, which has increased by 57% over the past 2 decades [1]. Surgery is the gold standard treatment for colorectal cancer, but adjuvant chemotherapy is often required because colorectal cancer has a recurrence rate of up to 30%. Recently, advances in chemotherapy have reduced the mortality and recurrence rates of colorectal cancer [2, 3]. Although chemotherapy increases survival rates, resistance to chemotherapy drugs has also increased. To date, the only effective chemotherapy for colorectal cancer is a combination of oxaliplatin, irinotecan, cetuximab, and bevacizumab, based on fluorouracil. Therefore, new cancer targets are needed.

Diabetes has been proposed as a risk factor for many cancers, including colon, breast, prostate, kidney, and pancreatic cancers [4, 5]. Insulin resistance in patients with diabetes can promote tumorigenesis by increasing the levels of insulin-like growth factor 1, steroidal sex hormones, and inflammation [6]. The first-line drug for patients with type 2 diabetes is metformin, which lowers insulin resistance. Recently, numerous studies have shown that metformin has anticancer effects [7, 8]. An early pilot case–control study investigating the effects of metformin in cancers associated with diabetes mellitus was published in 2005 [9]. Various mechanisms have been proposed for the anticancer effects of metformin. The induction of adenosine monophosphate-activated protein kinase (AMPK) is associated with multiple functions of metformin [10]. AMPK plays a critical role in maintaining cellular functions under energy-restricted conditions. Activated AMPK inhibits the synthesis of glucose, lipids, proteins, and cell growth under general conditions. AMPK activation inhibits mTOR signaling, subsequently inhibiting protein synthesis and cell proliferation, which may be a direct mechanism driving metformin-mediated suppression of cancer cell growth [11, 12]. Regulation of the mTOR pathway is considered the most important anticancer mechanism of metformin. Several other anticancer mechanisms have been proposed, including inhibition of serum insulin and insulin like growth factor 1 levels [13], downregulation of cyclin D1 protein expression [14], and activation of apoptotic pathways [15].

Although several mechanisms have been identified, it is necessary to determine other unknown antitumor mechanisms of metformin to identify appropriate cancer targets. High-throughput data has provided a new approach for understanding the pathophysiology of colorectal cancer. Analysis of gene expression patterns can reveal unknown disease etiologies of colon cancer [16]. Similarly, genetic association studies can facilitate the discovery of new mechanisms through which metformin influences colon cancer progression. In this study, we first performed a genetic association study to determine the mechanism of action of metformin in colorectal cancer. We found that metformin treatment reduced interleukin 6 (IL-6), inflammatory, and epithelial–mesenchymal transition (EMT) signaling. We then assessed these pathways *in vitro*.

## Materials and methods

### Gene expression and clinical datasets

In this study, we used public data to analyze mRNA expression. To identify the molecular changes in colon cancer cells upon treatment with metformin, we analyzed microarray data from LoVo cells treated with 10 mM metformin for 24 h (Gene Expression Omnibus accession no. GSE67342) [17]. LoVo cells treated with 10 mM of metformin were used as the metformin group, while untreated cells were used as the control group. Twenty-four hours later, control and treated cells were harvested for RNA extraction. We analyzed genes from the results of microarray with a *p*-value less than 0.001 (two-tailed Student’s *t*-test) and expression ratio greater than 1. The identified gene signatures were applied to the colon adenocarcinoma patient RNA sequencing dataset (Illumina HiSeq, San Diego, CA, USA) at TCGA with median centering. We also used the patient survival and stage data (TCGA), which was obtained from the cancer browser website (https://genome-cancer.ucsc.edu).

### Pathway analysis and analysis of the gene signature

The gene signature from metformin-treated LoVo cells was used to predict TCGA colon adenocarcinoma patients. A total of 329 patients were enrolled from TCGA for prediction. Predictions were using a bias-corrected hierarchical Bayesian classification method and TCGA patients were divided into a metformin-predicted group or control-predicted group based on a Bayesian probability of 0.5. We performed survival analysis for 205 patients who were in stage 1–3 for whose survival data had been recorded. We constructed a Kaplan–Meier survival curve and used the log-rank test to determine the differences between the 2 groups. For pathway analysis between the predicted groups, we performed core analysis using IPA software. The data used in this analysis included 1385 genes with a *p*-value of ≤ 0.001 as determined by Student’s *t*-test to compare the 2 groups. In addition, we applied GSEA to the dataset from TCGA with the hallmark gene set from the molecular signatures database [18]. The GSEA results were based on 6273 genes with a standard deviation greater than 1. According to the GSEA document, a false discovery rate lower than 0.25 was defined as significant.

### Cell culture and reagents

The human colon cancer cell lines HCT 116 and COLO 205 were purchased from the American Type Culture Collection (ATCC, Manassas, VA, USA) and maintained according to the manufacturer’s instructions. Recombinant human IL-6 was synthesized by PeproTech (Rocky Hill, NJ, USA). STAT3 and phosphorylated STAT3 were obtained from Cell Signaling Technology (Danvers, MA, USA). Metformin was obtained from Sigma-Aldrich (St. Louis, MO, USA).

### Cell viability assay

To determine the effects of IL-6 on the viability of colon cancer cells, we performed an MTT assay. The cells were seeded (1 × 10^4^ cells/well) in 96-well culture plates and then incubated for 24 h at 37°C in a 5% CO_2_ atmosphere. We changed the medium to Roswell Park Memorial Institute (RPMI)-1640 containing 2% fetal bovine serum with or without IL-6. After incubation for 24 or 72 h, the number of viable cells in duplicate wells was determined using the EZ-Cytox Cell Viability Assay kit (Daeil Lab Service, Seoul, Korea) according to the manufacturer’s instructions.

### Cell migration and Matrigel invasion assay

Colon cancer cells were seeded (5 × 10^5^ cells/well) in 12-well plates and then pre-incubated for 24 h in serum-free RPMI before a wound was created in the cell monolayer with a plastic pipette tip. The cells were grown in culture medium in the presence or absence of IL-6, with or without metformin. Cell migration across the wound was monitored by microscopy at 24 or 48 h. The ratio of the remaining wound area relative to the initial wound area was determined, and wound closure of the monolayer was calculated using Image-Pro Plus (Media Cybernetics, Rockville, MD, USA). The results are reported as percent wound closure. This assay was repeated independently 3 times.

Cells were seeded (1 × 10^5^ cells/well) in the upper chamber of a culture plate, which was coated with Matrigel. Serum-free medium containing IL-6 (50 ng/mL) or metformin (10 mM) was added to the lower chamber. After 24 h of incubation, non-migrating cells were removed from the upper chamber with a cotton swab, while cells on the lower surface of the insert were stained with hematoxylin and eosin. Invading cells were counted by microscopy using an imaging analysis program. All experiments were repeated 3 times.

### Western blotting

Equal quantities of protein were resolved by sodium dodecyl sulfate-polyacrylamide gel electrophoresis, followed by transfer to nitrocellulose membranes (Hybond-P; Amersham Biosciences, Piscataway, NJ, USA). The membranes were blocked with 5% non-fat dry milk in phosphate-buffered saline (PBS) containing 0.2% TWEEN 20 for 2 h and then incubated overnight at 4°C with primary antibodies against STAT3, phosphorylated STAT3, or β-actin (Sigma-Aldrich). The membranes were washed and incubated with horseradish peroxidase-conjugated secondary antibodies and the signals were detected with X-ray film. Densitometry was performed using ImageJ version 1.51 kit (NIH, Bethesda, MD, USA) after all western blotting.

### Confocal microscopy

Cells treated in the presence or absence of IL-6 (1, 5, or 10 ng/mL) for 24 h were fixed in 3.7% paraformaldehyde, permeabilized with 0.5% Triton X-100 for 10 min, and blocked with 3% bovine serum albumin for 30 min at room temperature. After blocking, the cells were incubated with primary antibodies overnight at 4°C and washed with PBS. The cells were incubated for 30 min at room temperature with a secondary Alexa Fluor 594- or fluorescein isothiocyanate-labeled secondary antibody (Molecular Probes, Eugene, OR, USA). The samples were co-stained with 2 μM 4′,6-diamidino-2-phenylindole (DAPI; Molecular Probes) and incubated at 37°C. After 3 washes with PBS, the slides were mounted using VECTASHIELD mounting medium (Vector Laboratories, Burlingame, CA, USA), and immunofluorescence was detected using a confocal microscope (Zeiss, Jena, Germany).

### qPCR

For qPCR, RNA (4 μg) was treated with DNase and reverse-transcribed into cDNA using an RnaUs Script reverse transcription kit (LeGene Biosciences, San Diego, CA, USA) according to the manufacturer’s protocol. Selected genes were analyzed by qPCR using the inventoried TaqMan assay (Applied Biosystems, Foster City, CA, USA), *MMP9* (HS00234579-m1) primers, and TaqMan master mix (Applied Biosystems) on the Applied Biosystems 7300 Real-Time PCR System. Expression of the target gene was normalized to that of glyceraldehyde 3-phosphate dehydrogenase (*GAPDH*, Hs99999905-m1) in the sample.

### Statistical analysis and institutional review board (IRB) approval

Statistical analyses were carried out using SPSS version 20.0 (SPSS, Inc., Chicago, IL, USA). ANOVA test and Turkey post-hoc analysis were used determined the statistical significance of the differences between the covariates. *p*-Values less than 0.05 were considered statistically significant. This research project was approved by the IRB of Korea University Guro Hospital (IRB no. KUGH16185-001).

## Results

### Colon cancer patients in the metformin-predicted group showed good clinical prognoses

**Fig 1:**
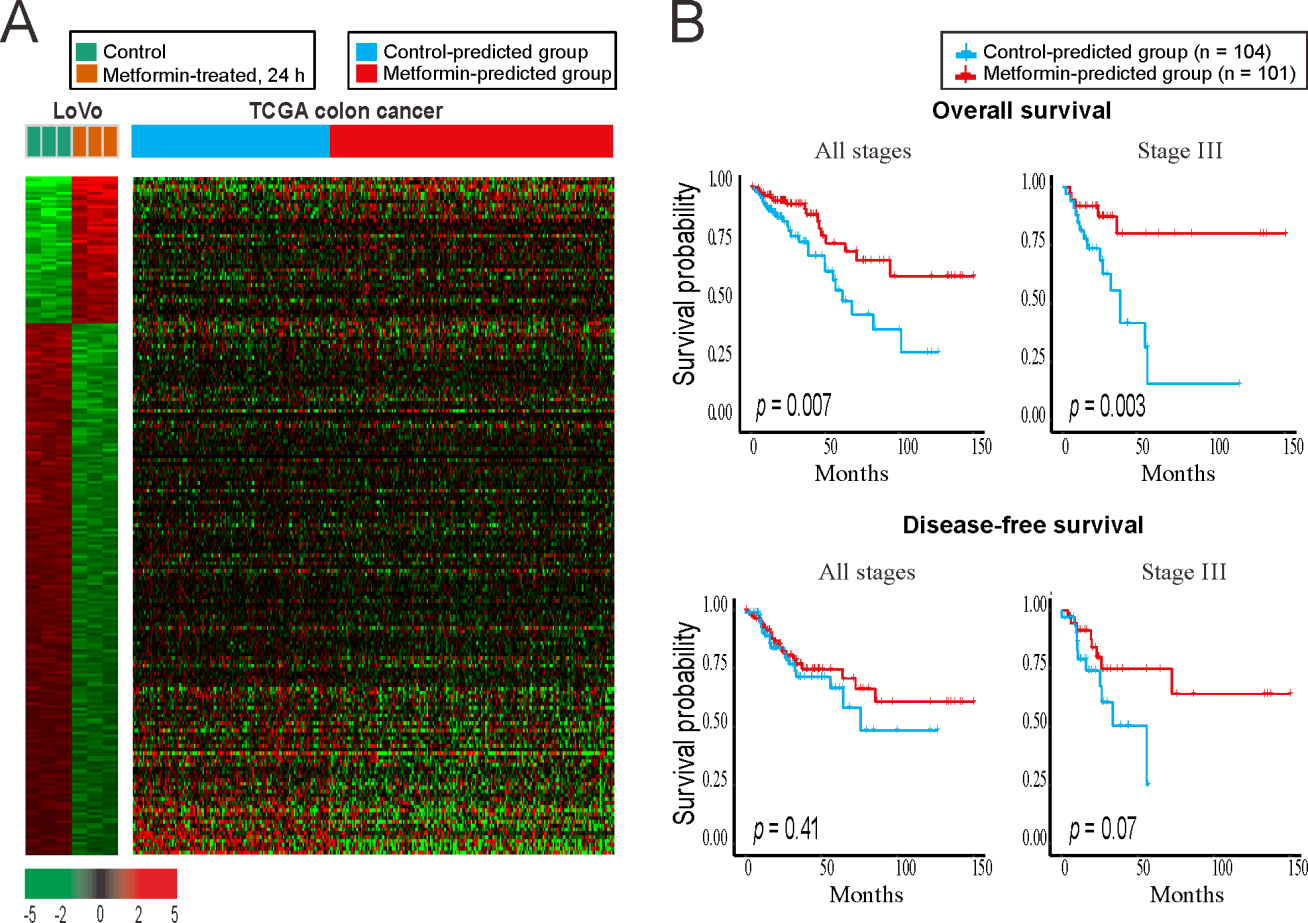
Gene expression profiles of colon cancer samples stratified by the metformin-treated gene signature of colon cancer cells (GSE67342). **(**A) Heatmap of the gene signature from the metformin-treated LoVo colon cancer cells and the control LoVo colon cancer cells (left). The prediction and grouping of TCGA colon cancer patients by the signature from the LoVo cells (right). (B) Kaplan–Meier survival plots of TCGA colon cancer patients by the results of prediction. The metformin-predicted group showed the better survival rate.

To investigate the changes caused by metformin in colon cancer, we analyzed the differences in mRNA expression between metformin-treated colon cancer cells and control cells. A total of 254 genes were selected as the metformin signature (S1 Table); these genes were statistically differentially expressed at the mRNA level in the group treated with metformin (10 mM) for 24 h compared to in control colon cancer cells (LoVo cells). To classify patients showing pattern of expression similar to the metformin signature, we analyzed the colon adenocarcinoma dataset from the Cancer Genome Atlas (TCGA) using a bias-corrected hierarchical Bayesian classification [19] method with the metformin signature (Fig 1). The patients were divided into 2 groups: TCGA patients were divided into the metformin-predicted group with a gene pattern similar to the metformin treatment group of LoVo cells and control-predicted group with a gene pattern similar to the control group of LoVo cells, although the patients were not directly treated with metformin. The metformin-predicted group consisted of 193 patients, while the control-predicted group had 136 patients (Fig 1A and S4 Table). We performed Kaplan-Meier analysis to compare the survival rate difference between the two groups in 205 patients with available survival data (Fig 1B). In survival analysis, the metformin-predicted group showed better survival than the control-predicted group at all stages (*p* = 0.007) and in stage III (*p* = 0.003). Recurrence-free survival was also higher in the metformin-predicted group, but the difference was not significant.

### Pathway analysis suggested that metformin reduces colon cancer metastasis by decreasing IL-6 signaling and preventing EMT

**Fig 2:**
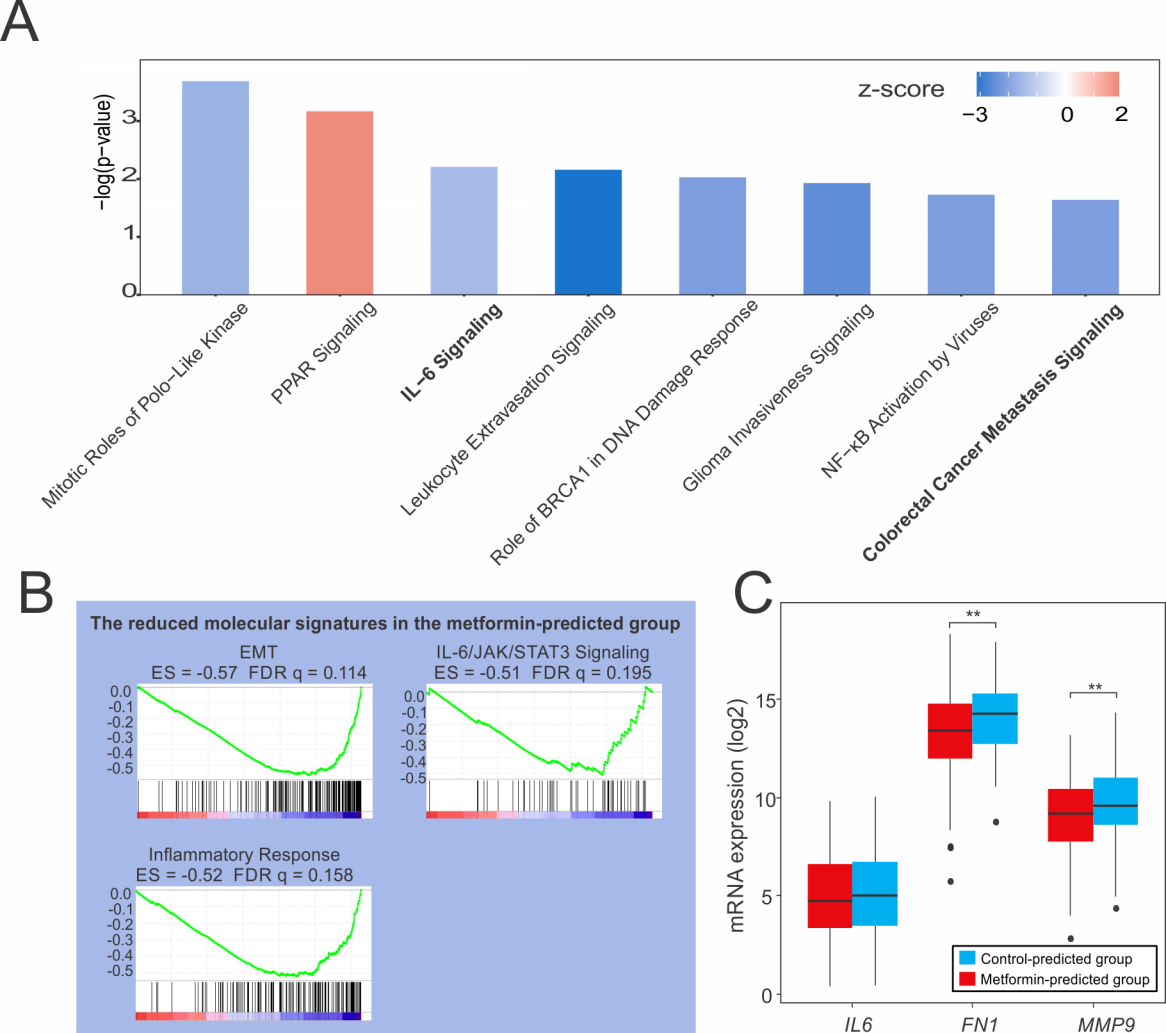
Pathway analysis of TCGA patients. **(**A) Canonical pathways in TCGA colon cancer patients as assessed by IPA. IL-6 and colorectal metastasis signaling were decreased. (B) GSEA showed that EMT and IL-6 signaling were reduced in the metformin-predicted group significantly. (C) mRNA expression of *IL6*, *FN1*, and *MMP9* was decreased in the metformin-predicted group (TCGA dataset). *p < 0.05, **p < 0.01, JAK: Janus kinase, ES: enrichment score, FDR: false discovery rate.

To identify pathways with differential activity in the 2 groups, we performed pathway analysis for the metformin-predicted group and control group identified in TCGA. First, we performed canonical pathway analysis with Ingenuity Pathway Analysis (IPA, Qiagen, Hilden, Germany) software. We filtered the results to select those with a -log(*p*-value) > 1.5 and z-score with an absolute value > 1.5 (S2 Table). The metformin-predicted group showed significant changes in 8 pathways (Fig 2A). Particularly, colon cancer metastasis signaling was significantly reduced (z-score, -2.132). In addition, IL-6 signaling, a type of inflammatory cytokine signaling that can influence cancer development and progression, was also reduced (z-score, -1.5) [20, 21]. Next, we applied gene set enrichment analysis (GSEA) to our TCGA dataset. When the hallmark gene set in MSigDB was applied, 18 pathways showed a false discovery rate of less than 25% (S3 Table). Among these pathways, the EMT pathway was significantly reduced to an enrichment score (ES) of -0.57 (Fig 2B). In addition, IL-6 signaling was decreased in the GSEA, as in the results of IPA, and inflammatory responses were decreased. Based on the results of pathway analyses, we confirmed the expression of representative genes from the reduced pathways identified in our TCGA dataset. Fibronectin (FN1) and matrix metallopeptidase 9 (MMP9) are markers for EMT and metastasis, respectively [22, 23]. In our TCGA data, the *FN1*, *MMP9*, and *IL6* transcripts were expressed at lower levels in the metformin-predicted group than in the control-predicted group (*FN1*, *p* = 0.001 and *MMP9*, *p* = 0.002) (Fig 2C). Zhao et al. [24] recently reported that IL-6 induces EMT through signal transducer and activator of transcription 3 (STAT3) phosphorylation, and that this process can be blocked in lung cancer by metformin. Our pathway analysis showed that metformin reduced IL-6 signaling, EMT, and colon cancer metastasis signaling; thus, we hypothesized that IL-6 also causes EMT in colon cancer, which may be inhibited by metformin. To test this hypothesis, we performed experiments in colon cancer cell lines.

### IL-6 induced EMT through STAT3 phosphorylation in colorectal cancer cells

**Fig 3:**
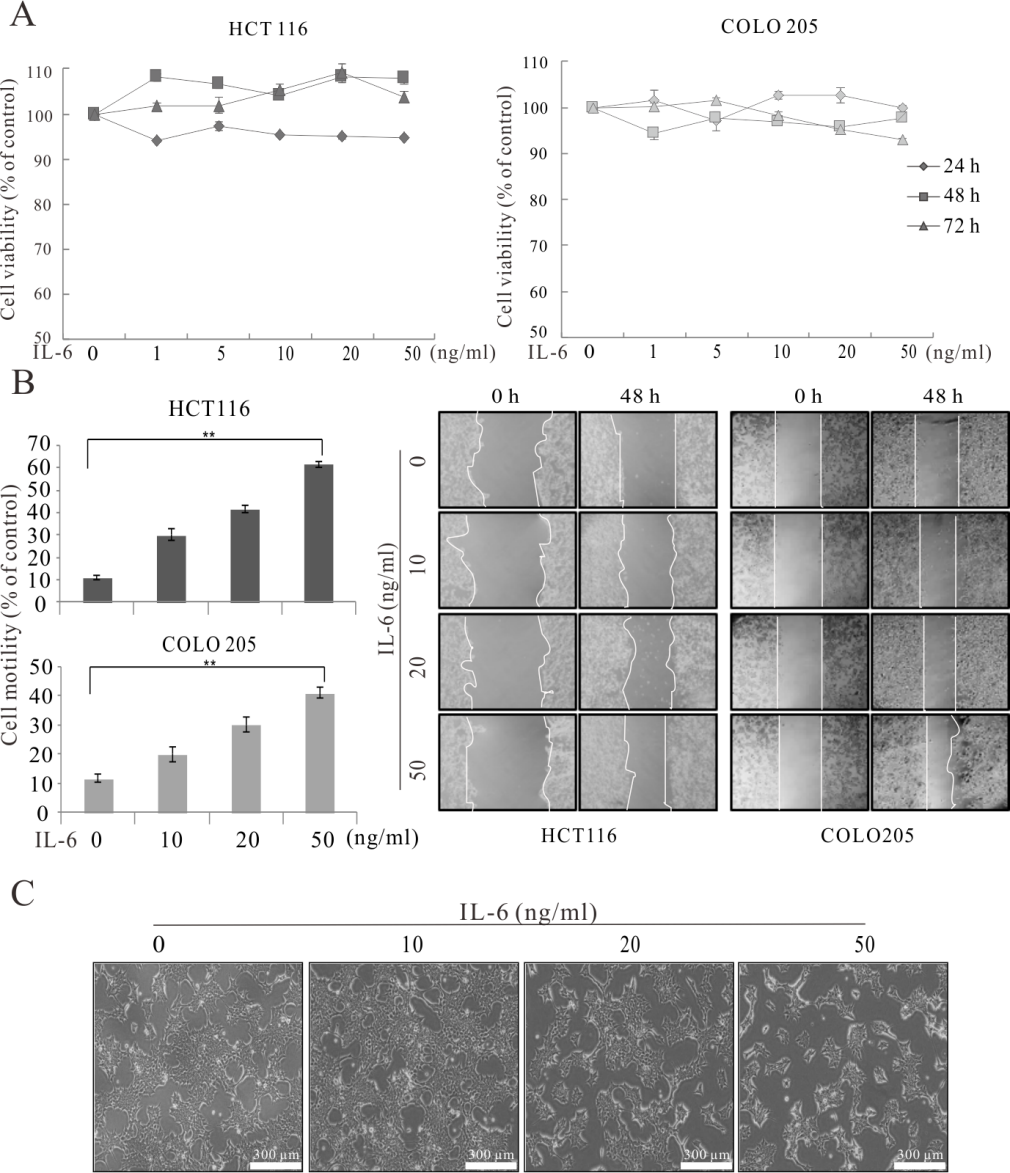
IL-6 induced EMT in colon cancer cells. (A) Cell viability assay for colon cancer cell lines. (B) Migration assay for colon cancer cell lines treated with different concentrations of IL-6 for the indicated times. (C) Morphological changes in colon cancer cells upon treatment with IL-6 at the indicated concentrations. Scale bar = 300 μm, HCT 116. *p < 0.05, **p < 0.01.

IL-6 is known to be involved in the metastasis in several cancers [25–27], and IL-6 may enhance cancer cell migration to promote metastasis in colon cancer. We first performed a methyl thiazolyl tetrazolium (MTT) assay to determine the effect of IL-6 on the viability of 2 colon cancer cell lines, HCT 116 and COLO 205. We treated the cells with various concentrations (1, 5, 10, 20, and 50 ng/mL) of IL-6 for different times, and observed no significant effects on cell viability (Fig 3A). To examine the effects of IL-6 on cancer progression, we conducted a migration assay. We treated HCT 116 and COLO 205 cells with IL-6 (10, 20, and 50 ng/mL) for 0 and 48 h. IL-6 treatment increased cell motility in a concentration-dependent manner (Fig 3B and S1 File). We also observed morphological changes in the cancer cells following IL-6 treatment (Fig 3C). After treatment, the cells changed from a clustered, cobblestone morphology to a mesenchymal-like morphology with scattered, polarized, spindle-shaped cells. These morphological changes were dose-dependent.

**Fig 4:**
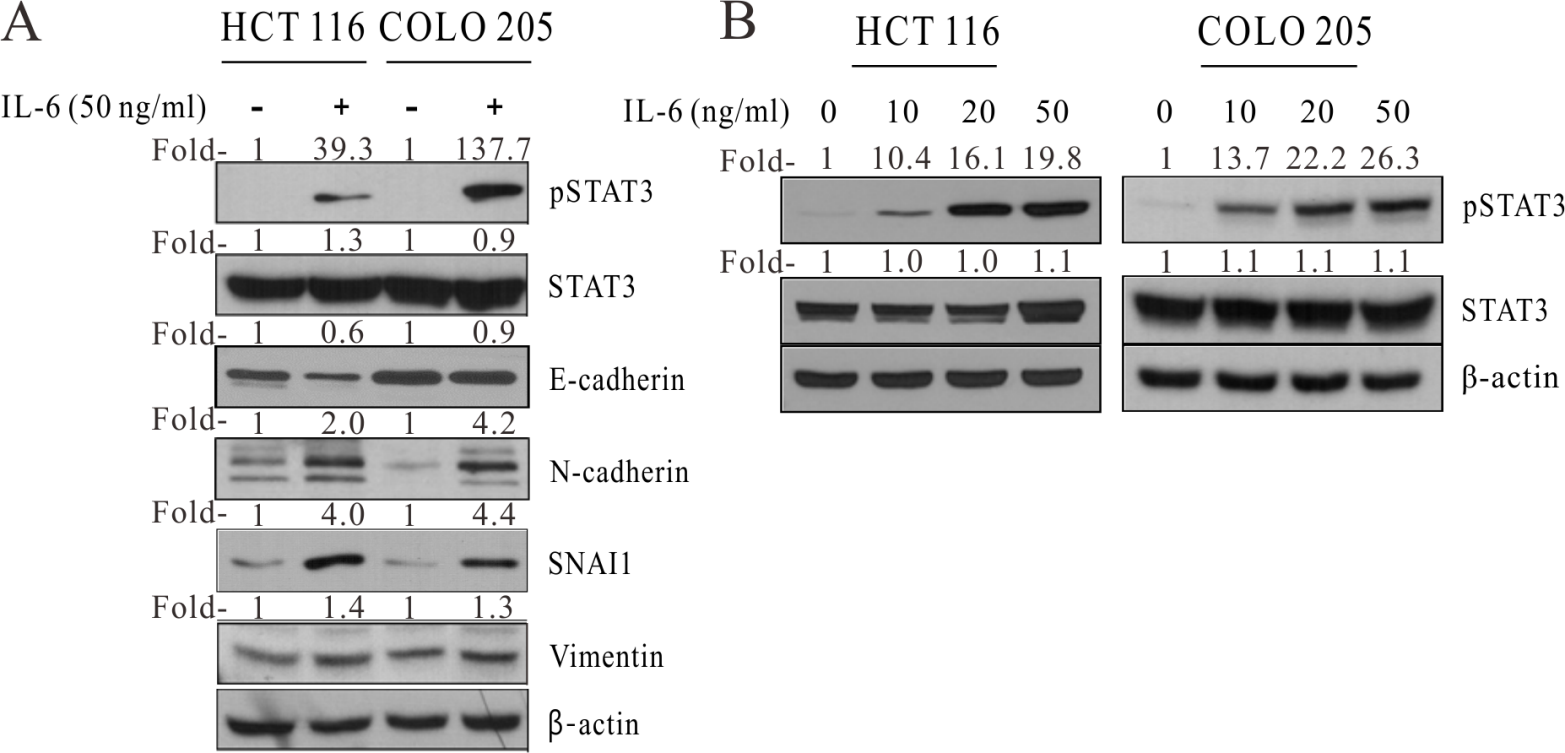
IL-6 induced EMT through STAT3 phosphorylation. (A) Western blot of HCT 116 and COLO 205 cells treated with 50 ng/mL IL-6. (B) Western blot analysis of HCT 116 and COLO 205 cells after exposure to increasing concentrations of IL-6. Fold-, fold-change; pSTAT3, phosphorylated STAT3; β-actin, beta actin.

Next, we treated HCT 116 and COLO 205 cells with 50 ng/mL IL-6 and analyzed protein expression by western blotting (Fig 4A). Phosphorylated STAT3 is known to be involved in EMT induction [28]. E-Cadherin (cadherin 1) is an epithelial marker, whereas N-cadherin (cadherin 2), vimentin, and snail family transcriptional repressor 1 (SNAI1) are well-known mesenchymal markers. Upon treatment with IL-6, the 2 colon cancer cell lines showed decreased levels of E-cadherin and increased levels of phosphorylated STAT3, N-cadherin, SNAI1, and vimentin. To determine the IL-6 dose dependency of EMT, HCT 116 and COLO 205 cells were treated with various doses of IL-6 (Fig 4B). We found greater dose-dependent STAT3 phosphorylation in cells exposed to higher concentrations of IL-6.

### Metformin reversed IL-6-induced EMT

**Fig 5:**
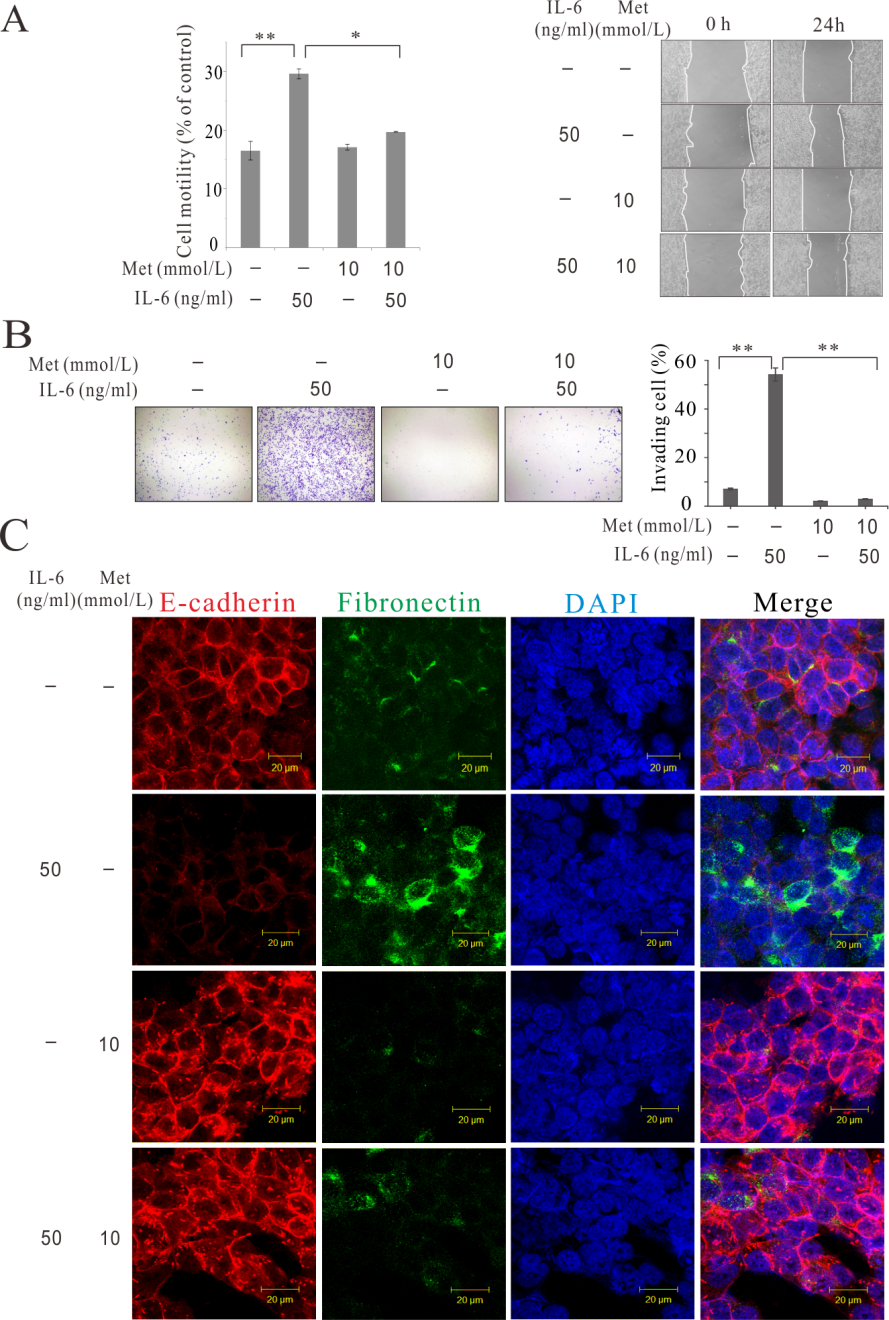
Metformin inhibited IL-6-induced EMT in the human colon cancer cell line. (A) Migration assay for colon cancer cells incubated with IL-6 and metformin (HCT116). (B) Invasion assay for colon cancer cells (HCT 116). (C) Immunofluorescence confocal microscopy analysis of HCT 116 colon cancer cells treated with IL-6 and metformin. Met, metformin. *p < 0.05, **p < 0.01.

We investigated whether metformin treatment suppresses IL-6-induced EMT in colorectal cancer cells. To determine the effect of metformin on IL-6-induced motility changes in colon cancer cells, we performed a migration assay (Fig 5A and S1 File). When HCT 116 cells were treated with 50 ng/mL IL-6, their motility increased, as demonstrated in the previous assay. Surprisingly, however, when HCT 116 cells were treated with IL-6 and metformin simultaneously, the increase in motility was significantly diminished. The greatest motility was observed in the IL-6 alone treatment group. We then investigated invasiveness, which is essential for metastasis. IL-6 enhanced the invasiveness of colon cancer cells, whereas metformin blocked their invasiveness (Fig 5B), which agrees with the results of the migration assay. Analysis of HCT 116 cells by immunofluorescence confocal microscopy revealed that IL-6 treatment decreased the expression of E-cadherin and increased the expression of fibronectin. However, simultaneous treatment with IL-6 and metformin increased the expression of E-cadherin and decreased the expression of fibronectin (Fig 5C).

**Fig 6:**
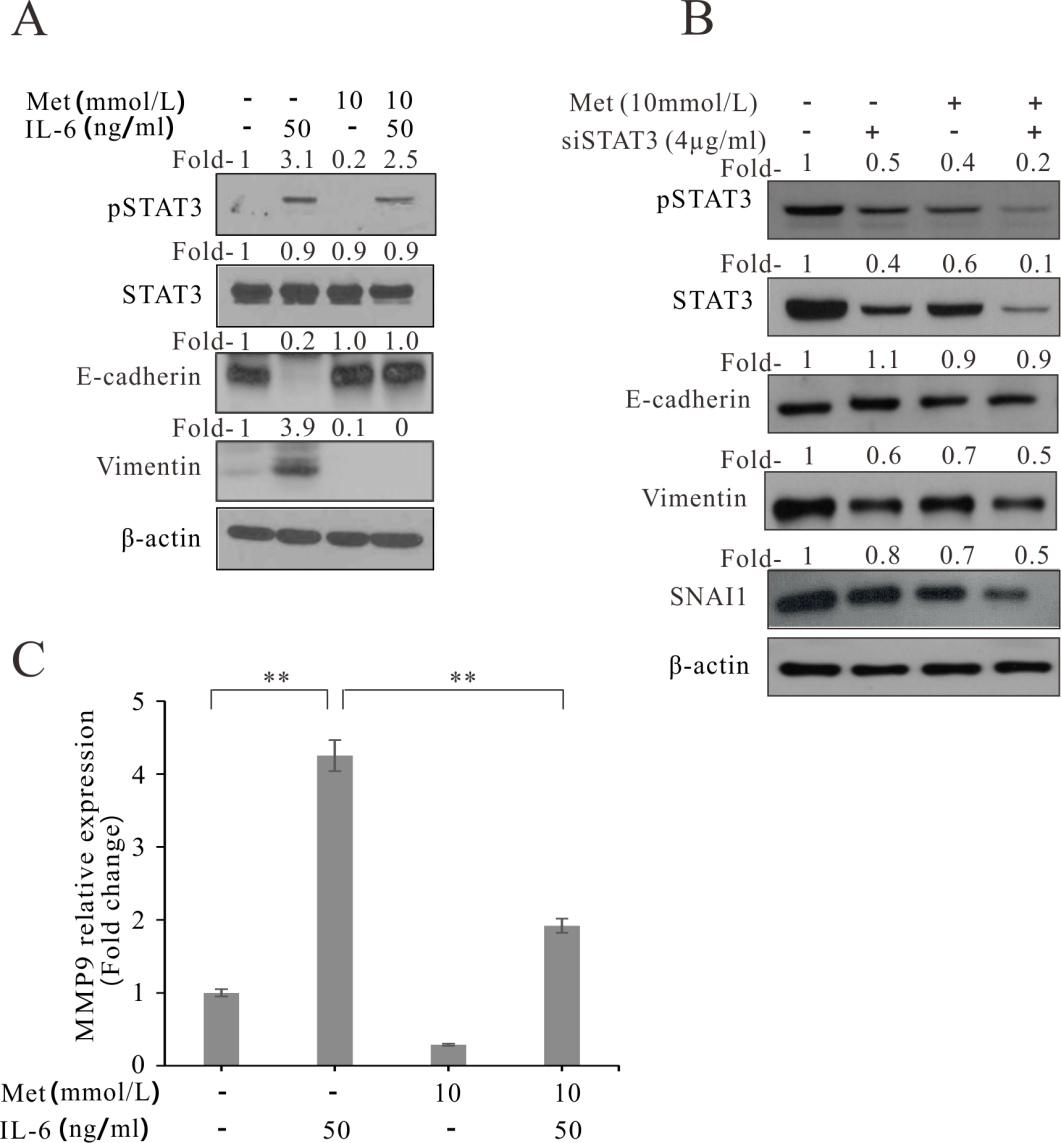
Effect of metformin on IL-6-induced STAT3 phosphorylation and *MMP9* expression in the human colon cancer cell line COLO 205. (A) Western blot analysis of metformin- and IL-6- treated colon cancer cells (HCT 116). (B) Western blot analysis of metformin- and siRNA STAT3 (siSTAT3)—treated colon cancer cells (HCT 116) (C) qPCR analysis of metformin- and IL-6-treated colon cancer cells. Fold-, fold-change; Met, metformin. *p < 0.05, **p < 0.01.

We also confirmed that phosphorylation of STAT3 by IL-6 was reversed by metformin. In western blot analysis, IL-6-induced phosphorylated STAT3 signals were faint in the metformin-treated colon cancer cell lines (Fig 6A). Following treatment with IL-6, E-Cadherin was decreased and vimentin was increased; these effects were reversed by treatment with metformin and IL-6. Furthermore, to confirm the relationship of STAT3 to EMT, siRNA-STAT3 (siSTAT3) was treated. The inhibition of STAT3 decreased the EMT markers, vimentin and SNAI1. And, treatment of siSTAT3 with metformin decreased vimentin and SNAI1 more than siSTAT3 treatment alone (Fig 6B). We also evaluated the effects of IL-6 and metformin on metastasis. MMP9, which plays a role in extracellular matrix remodeling and angiogenesis, is also associated with cancer metastasis [23]. To quantitatively analyze the association of IL-6 and metformin with cancer metastasis, we measured the expression of *MMP9* by real-time polymerase chain reaction (qPCR). As expected, IL-6 enhanced the expression of *MMP9*, while metformin reduced the IL-6-enhanced expression of *MMP9* in colon cancer cells (Fig 6C).

## Discussion

IL-6 is a multifunctional cytokine that plays an important role in mediating inflammation and immune responses. Recently, it has also been reported that IL-6 is an important mediator of the tumor-promoting effect of inflammation-related diseases, and that patients with advanced or metastatic colon cancer have elevated serum IL-6 levels [29]. Nagasaki et al. reported that the source of IL-6 in cancer can be stromal cells rather than tumor cells. Cancer stromal fibroblasts play an important role in IL-6 production and cancer progression [30]. Generally, the inflammation induced by IL-6 is a physiological process that is essential for removing harmful stimuli and initiating healing. However, it also plays a key role in the pathogenesis of many cancers. For example, inflammatory bowel disease is a well-known risk factor for colorectal cancer, and approximately 25% of cancers are associated with chronic infection or chronic inflammation [31]. Although solid tumors are not always associated with chronic inflammation, an inflammatory microenvironment has been shown to be involved in tumorigenesis [32]. Grivennikov et al. reported that the incidence and progression of colorectal cancer are driven by inflammation-induced barrier defects [33]. Bollrath et al. suggested that STAT3 is required for the growth of colitis-associated colorectal cancer in mouse models [34]. We showed that IL-6 induced EMT, which promotes colon cancer progression, and that STAT3 phosphorylation was involved in this pathway. Colon cancer cell migration and invasion were activated by IL-6 and the expression of EMT markers was increased in cells treated with IL-6. In addition, increasing concentrations of IL-6 caused dose-dependent increases in STAT3 phosphorylation.

Metformin, the first-line treatment for type 2 diabetes [35], has recently attracted attention for its anticancer effects. The antitumor mechanism mainly occurs via the mTOR pathway [36]. In the present study, IPA analysis revealed that the mTOR pathway had a *p* value of 0.485 and z-score of -0.707, which were not significant (S2 Table). However, GSEA analysis revealed that the mTOR pathway of the metformin-predicted group showed the most significant signal increase with a false discovery rate of 0.009 and normalized enrichment score of -2.136 (S3 Table). mTOR signaling is a key factor in the metformin mechanism according to pathway analysis in this study. However, our goal was to identify a new mechanism other than the mTOR pathway. Therefore, we focused on IL-6 signaling and EMT.

In addition to the mTOR pathway, IL-6 signaling and EMT were found to be altered by metformin treatment in our analysis. Notably, recent reports showed that EMT and STAT3 signaling are involved in the antitumor mechanism of metformin. Lind et al. [37] reported that metformin inhibits growth and activates the apoptosis of triple-negative breast cancer cells by preventing STAT3 phosphorylation. Another group showed that metformin decreases EMT in breast cancer cells by diminishing key factors, such as zinc finger E-box binding homeobox 1, TWIST1, and snail family transcriptional repressor 2 [38]. In addition, metformin can reduce IL-6-induced EMT in lung cancer patients [24]. However, this effect has not been investigated in colorectal cancer. In our TCGA gene dataset for colorectal cancer, we found that IL-6, EMT, and inflammation signaling were decreased in the metformin-predicted group. This finding suggests that metformin reduces the effect of IL-6 on EMT in colorectal cancer, as well as in cancers reported in previous studies. In addition, metformin decreased IL-6-induced increases in cell motility, invasiveness, and MMP9 expression. STAT3 phosphorylation induced by IL-6 was also blocked by metformin. This is the first *in vitro* study to demonstrate the EMT-suppressive function of metformin in IL-6-induced colon cancer. Although this study has been derived from human datasets, further *in vivo* studies are required to support our *in vitro* results.

In conclusion, genomic data analysis revealed that metformin can reduce IL-6 signaling, EMT, and colon cancer metastasis. These findings were confirmed by *in vitro* experiments. We found that IL-6 induced EMT in colon cancer cells and that STAT3 phosphorylation was involved in this process. We also found that metformin inhibited IL-6-induced EMT by inhibiting STAT3 phosphorylation.

## Supporting information

S1 Table The metformin signature. The gene signature extracted from metformin-treated LoVo cells.
(XLSX)

S2 Table IPA anlaysis for canonical pathway. (XLSX)

S3 Table The gene set enrichment analysis result. (XLSX)

S4 Table The demographics of colon cancer patients. (XLSX)

S1 File The tables for statistics analysis. (XLSX)
